# Urbanization increases floral specialization of pollinators

**DOI:** 10.1002/ece3.8619

**Published:** 2022-03-07

**Authors:** Sevan Suni, Erin Hall, Evangelina Bahu, Hannah Hayes

**Affiliations:** ^1^ 7149 Department of Biology University of San Francisco San Francisco California USA

**Keywords:** foraging, frequency‐dependent, habitat fragments, invasive plant, pollen, pollinator, specialization, urbanization

## Abstract

Understanding how urbanization alters functional interactions among pollinators and plants is critically important given increasing anthropogenic land use and declines in pollinator populations. Pollinators often exhibit short‐term specialization and visit plants of the same species during one foraging trip. This facilitates plant receipt of conspecific pollen—pollen on a pollinator that is the same species as the plant on which the pollinator was foraging. Conspecific pollen receipt facilitates plant reproductive success and is thus important to plant and pollinator persistence. We investigated how urbanization affects short‐term specialization of insect pollinators by examining pollen loads on insects’ bodies and identifying the number and species of pollen grains on insects caught in urban habitat fragments and natural areas. We assessed possible drivers of differences between urban and natural areas, including frequency dependence in foraging, species richness and diversity of the plant and pollinator communities, floral abundance, and the presence of invasive plant species. Pollinators were more specialized in urban fragments than in natural areas, despite no differences in the species richness of plant communities across site types. These differences were likely driven by higher specialization of common pollinators, which were more abundant in urban sites. In addition, pollinators preferred to forage on invasive plants at urban sites and native plants at natural sites. Our findings reveal indirect effects of urbanization on pollinator fidelity to individual plant species and have implications for the maintenance of plant species diversity in small habitat fragments. Higher preference of pollinators for invasive plants at urban sites suggests that native species may receive fewer visits by pollinators. Therefore, native plant species diversity may decline in urban sites without continued augmentation of urban flora or removal of invasive species.

## Graphical abstract

Short‐term foraging specialization of pollinators on individual plant species can facilitate plant reproductive success. We asked if short‐term specialization changed in urban environments, and we found that pollinators were more specialized in urban areas. This pattern was driven by higher affinity of common pollinators to individual plant species in urban areas.

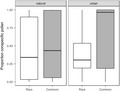

## 1 INTRODUCTION

Over 50% of the Earth's land surface has been converted for human use (Ritchie & Roser, 2017). Given recently observed declines in pollinator populations (Goulson et al., 2015), understanding links between urbanization and plant–pollinator interactions is of increasing importance. Almost 90% of flowering plants are animal‐pollinated (Ollerton et al., 2011), and one‐third of crops require pollinators to produce fruit (Kearns et al., 1998). Declines have been reported in all major groups of pollinators (Regan et al., 2015), which are associated with declines in plant populations (Biesmeijer et al., 2006). While land conversion from natural to urban areas has been proposed as a major driver of pollinator decline (Bates et al., 2011; Forister et al., 2019; Hernandez et al., 2009), other work has challenged the assumption that urbanization is universally detrimental to pollinators (Baldock et al., 2015; Owen, 2010; Saure, 1996). Effects of urbanization tend to be species‐specific, with some species increasing and others decreasing in abundance in urban areas (Cane et al., 2006; Carre et al., 2009; Matteson et al., 2008). This may reflect differences in the ability of individuals of certain species to exploit patchy urban floral resources, or a lack of continuously blooming flowers that result in insufficient food intake of pollinators with certain phenologies. Declines in plant populations in urban areas are also well documented, and can stem from reproductive dependence of plants on pollinators and lower pollinator abundance in urban areas (Aguilar et al., 2006). In addition, effects of invasive plants on native plants may drive plant declines. Invasive plants are common in urban areas, and competition with native taxa for pollinators can lower seed set of native plants (Brown et al., 2002).

Given ongoing land conversion, remnant or restored natural habitat within urban areas will be increasingly important refuges of plant and pollinator biodiversity (Goddard et al., 2010). The extent to which habitat fragments can support pollinators depends on whether there are sufficient floral resources from which pollinators can obtain food, and whether these resources persist over time. In turn, persistence of plant populations depends on receipt of pollen from conspecific individuals, which depends directly on pollinator foraging choices. Floral specialist pollinators visit the same plant species on consecutive visits (Müller, 1996), and can be effective pollinators of plants with generalized pollination systems (Larsson, 2005; Parker, 1981; but see Neff & Rozen, 1995). However, urbanization tends to shift species assemblages toward a higher proportion of generalist pollinators (Deguines et al., 2016), which may visit different plant species over their lifetimes or within a single foraging trip. Generalist pollinators are expected to maximize their net energy intake while foraging (Stephens & Krebs, 1986), and their foraging choices depend on the distribution of floral resources, the energetic value of those resources, and the local ecological context (MacArthur & Pianka, 1966). Variation among generalist pollinators in the amount of pollen carried and deposited, visitation rates, and propensity to transfer self‐pollen have been documented (Földesi et al., 2021; Ivey et al., 2003). For example, hyperabundant honeybees (*Apis mellifera*) can have higher visitation rates than other species, but increase rates of self‐pollen transfer relative to other bee species, which decreases seed set (Sun et al., 2013). Therefore, plant reproductive success in urban fragments should depend on which pollinator species become dominant, as well as the extent to which population sizes of specialist pollinators are reduced.

Foraging choices of generalist pollinators may promote the persistence of outcrossing plants if ecological conditions facilitate the transfer of conspecific pollen among plant individuals (Aguilar & Galetto, 2004). Plant receipt of conspecific pollen is facilitated by short‐term specialization of those pollinators on that particular plant species. Several non‐mutually exclusive mechanisms can drive short‐term specialization, including flower constancy and frequency dependence in plant choice. Flower constancy occurs when pollinators forage primarily on the same plant species within a single trip (Waser, 1986; Wissel, 1977), which can be facilitated by interspecific competition (Brosi & Briggs, 2013; Futuyma & Moreno, 1988). Decreases in flower constancy lead to greater heterospecific pollen transfer among plants and reduced plant reproductive success (Brosi & Briggs, 2013; Galen & Gregory, 1989). Urbanization is associated with lower pollinator species richness (Bates et al., 2011), leading to the prediction that it should reduce interspecific competition and thus also conspecific pollen transfer. However, diverse pollinator assemblages are sometimes found in urban areas (Baldock et al., 2019). Therefore, predicting competition‐mediated effects of urbanization on short‐term specialization is not straightforward, and may require a nuanced understanding of other ecological aspects of the specific geographic area.

Frequency dependence in plant choice occurs when there is a relationship between plant relative abundance and pollinator preference for that species (Krebs et al., 1989). Positive frequency dependence is predicted by optimal diet theory (MacArthur & Pianka, 1966) and is thought to occur due to difficulties in efficiently foraging on multiple floral types consecutively (Chittka & Thomson, 1997). Negative frequency dependence in plant choice is predicted if common flowers tend to be rewardless (Smithson & McNair, 1997), or if niche partitioning due to interspecific competition occurs such that common species forage preferentially on common resources and rare species forage preferentially on rarer resources (Eckhart et al., 2006; Possingham, 1992). While both positive and negative frequency dependence in plant choice can drive short‐term specialization, they may have different effects on plant species persistence in small habitat fragments. Negative frequency dependence is associated with species coexistence, as preference for rare morphs facilitates their reproductive success and thus persistence. In contrast, positive frequency dependence is associated with the decline of rare species, as preference for common morphs leads to common morphs becoming more common and rare morphs becoming more rare (Chesson, 2000, but see Molofsky & Bever, 2002).

Here, we ask how short‐term ecological specialization of pollinators may change in urban environments. We first characterize the visitation of pollinators to plants and pollen loads on pollinators’ bodies, and we quantify the amount of conspecific pollen relative to heterospecific pollen carried by pollinators in natural and urban sites across a major urban area in California. We then examine the possible direct and indirect drivers of difference among sites including whether sites were in urban or natural areas, aspects of the plant and pollinator communities at each site, pollinator sex, pollinator rarity, invasive plant presence, and frequency‐dependent foraging of pollinators. We find indirect effects of urbanization on short‐term specialization of pollinators that are mediated by pollinator rarity. We also find the indirect effects of invasive plants on short‐term specialization that are mediated by the amount of pollen carried by pollinators. Our analysis of biotic and abiotic drivers of short‐term pollinator specialization in urban and natural areas provides insights into mechanisms that may mediate effects of urbanization on plant reproductive success, plant species coexistence, and pollinator persistence in urban fragments.

## 2 METHODS

### 2.1 Site characterization

From May to August 2019, we characterized interactions between insect pollinators and plants at six natural and six urban sites in the Bay Area of California, United States (Figure S1). Urban sites were sites embedded within city limits and contained either remnant or restored natural habitat (Table 1). Sites were designated as urban or natural using data available at https://www.bayarealands.org/maps‐data along with imagery from Google Earth. Between the two points furthest from one another along the perimeter, urban sites spanned less than 4 km, and natural areas spanned at least 60 km (see Figure S1). The mean percent impervious surface, calculated using the National Land Cover Database (Homer et al., 2007) in ArcGIS Pro v2.8.1 (Esri, USA), from a 4‐km radius buffer around each site, was 21 ± 4% for natural sites and 59 ± 3.5% for urban sites (see Table 1 for values for each site).

**TABLE 1:** For each site, whether it was embedded within an urban area (U) or was part of a large tact of natural land (N), the date sampled, the % impervious surface of a circle of radius 8 km surrounding each sampling location (% IS), the number of pollinator samples obtained (N), the species richness (SR) and exponentiated Shannon Diversity (SD) of plant species growing at sites as assessed with transects, the species richness of invasive plants on transects (Inv. SR), the number of pollinators that were foraging on invasive plants (Inv. for.), the species richness (SR) and exponentiated Shannon Diversity (SD) of pollinator species and plant species from which pollen was found on pollinators (averaged over pollinators), and the total number of plant species from which pollen was found across all pollinators (tot). The bottom row shows species richness of each category across all sites

Site	Type	Date	% IS	N	Plant SR	Plant SD	Inv. SR	Inv. for.	Insect SR	Insect SD	Pollen SR (avg.)	Pollen SD (avg.)	Pollen SR (tot.)
AZ	N	5/22/19	6.6	46	6	3.8	0	0	6	3.7	3.1	0.58	16
BO	N	5/30/19	13.7	15	8	5.9	5	9	5	4.1	2.5	0.54	11
HH	U	8/8/19	27.2	48	11	8.1	2	0	2	2.0	2.2	0.42	6
LE	U	5/28/19	59.6	36	4	2.5	1	36	7	3.6	1.9	0.35	15
LM	U	6/4/19	65.7	49	4	2.4	2	6	3	2.1	2.4	0.49	16
MB	N	6/17/19	27.3	48	6	3.4	5	42	9	3.2	2.2	0.64	8
MH	N	6/10/19	23.8	24	4	2.6	3	32	5	3.1	2.7	0.63	9
MS	U	6/26/19	65.0	49	7	2.6	3	1	3	1.4	2.2	0.40	14
PO	U	7/10/19	58.3	47	2	2.0	1	24	1	1	1.7	0.48	7
RM	U	8/14/19	41.8	33	2	1.1	0	0	2	1.1	1.4	0.06	3
RS	N	6/12/19	18.9	49	6	2.8	5	5	11	7.3	2.1	0.34	13
SR	N	6/5/19	37.3	49	8	4.8	7	32	10	4.3	2.0	0.51	15
Total	–	–	–	493	43	–	17	12	27	–	–	–	56

### 2.2 Field sampling & community‐level metrics

Between 10 am and 4 pm, we used areal netting to haphazardly catch an average of 41.1 foraging insects per site (range 15–49). We targeted non‐Lepidopteran insects, and obtained 493 insects in total (Table 1). Each insect was placed into its own individual vial, and transported back to the laboratory for pollen removal. For each plant on which a pollinator was caught, we recorded the species and sampled three anthers from one flower. At each site, we also counted and recorded the species of all flowers within 15 cm of a 90‐m transect, and we sampled anthers from the other flowering plants. We determined if each plant in transects or on which a pollinator was caught is invasive in California using *the California Invasive Plant Council Dataset*, as well as the *Calflora Database*. Plants were considered invasive if they were both non‐native and were documented to competitively dominate some native communities. For each site, we characterized plant and pollinator species richness, and we calculated species diversity using exponentiated Shannon Diversity as in Chao et al. (2014).

### 2.3 Pollen load assessment

We removed pollen from pollinators’ bodies and corbicula with Fuchsin jelly (Kearns & Inouye, 1993). For each pollinator, we melted the jelly onto a slide (hereafter “pollinator slides”), homogenized the pollen in the melted jelly using sterilized tweezers, and visualized pollen using a light microscope. We also made slides of pollen from the anthers of the plant species on which each pollinator was caught (hereafter “anther slides”). For each pollinator, we compared the pollen on the anther slide to the pollen on the pollinator slide, and considered pollen grains conspecific if they were morphologically identical to those from the plant on which the pollinator was caught. Pollen from different species may look similar under a light microscope. To mitigate against the possibility of similar looking pollen confounding assessments of the proportion of conspecific pollen, we also made pollen slides from the anthers of other flowering plants at each site to determine if pollen from other species was similar in appearance, and also to determine from which species heterospecific pollen came. To identify heterospecific pollen to species, we compared pollen on pollinator slides to pollen made from the anthers of other flowering plants at each site. We cross‐referenced pollen identifications with photos from the Global Pollen Project (globalpollenproject.org). Floral diversity was relatively low across sites (see Results), and pollen from plants in the community was distinct enough to distinguish conspecific from heterospecific pollen on slides for all sites.

We examined the potential for conspecific pollen transfer as follows. For each pollinator, we counted the number of pollen grains that were morphologically identical to grains obtained from the anther slide on which the pollinator was caught (hereafter “conspecific pollen grains”), from a subsample of 500 pollen grains. To do so, we began examining each slide at the upper left corner, and we recorded the species of each grain until either 500 grains were encountered (*N* = 355 slides) or all the pollen on the slide had been counted (*N* = 158 slides; Table S1). We asked how different our estimate of the proportion of conspecific grains from a subsample of 500 might be from the proportion calculated using all grains. To do so, we randomly selected 10 pollinator slides that had more than 500 total grains and counted and identified all grains (59,189 grains). The correlation of the proportion of conspecific grains estimated from the subsample of 500 grains and that estimated from all grains was 0.87 (Pearson correlation, two‐sided test, *t* = 5.1, *p* < .001).

### 2.4 Pollinator rarity and species‐level specialization

We evaluated if the abundance of individual insect species differed among sites. Most insect species were found at five or fewer sites, with the exception of one honeybee and one bumble bee species (*Apis mellifera* and *Bombus vosnesenskii*) that were found at 10 and 11 sites, respectively. *Apis mellifera* and *B*. *vosnesenskii* were considered “common” pollinators in models (see below), while the remaining insect species were considered “rare” pollinators. We quantified species‐level floral specialization of pollinators at each site by constructing a flower interaction network from the pollen found on slides. We estimated floral specialization using the statistic *d’*, which we calculated using the R package Bipartite (Dormann, 2011). This statistic measures how specialized a species is with respect to available resources.

### 2.5 Frequency‐dependent foraging

To evaluate if pollinators exhibit frequency dependence in visitation, we asked if plant relative abundance predicted a metric that reflects pollinator preference (hereafter PI). We calculated PI for each species *s* at each sampling location *p* as in Grüter et al. (2011):

$${\text{PI}}_{s,p}=\frac{P_{\text{obs}\;s,p}}{\left(P_{\text{obs}\;s,p}+P_{\text{null}\;s,p}\right)},$$

where $P_{\text{obs}\;s,p}$ is the proportion of pollen grains found on pollinators at site *p* that are species *s*, and $P_{\text{null}\;s,p}$ is the proportion of flowers of focal plants of species *s* among flowers of all plants in the transect. A PI value of 0 indicates no pollen from species *s* was found on pollinators at site *p*, a value of 0.5 indicates that the observed pollen amount matches expectations based on plant relative abundance, and ${\text{PI}}_{s,p}$ approaches 1 if the amount of pollen from species *s* is much higher than that based on plant relative abundance. For these calculations, we used a dataset that included plant species that were present in transects.

### 2.6 Modeling drivers of short‐term specialization

We fitted three piecewise structural equation models (SEMs) to test the direct and indirect effects of landscape variables and aspects of the plant and pollinator communities on short‐term specialization, using the PiecewiseSEM R package (Lefcheck, 2016). An advantage of SEMs is that they allow for tests of direct and indirect effects of networks of potentially correlated variables on response variables of interest. We fitted one SEM for each of the following measures of short‐term specialization: the proportion of conspecific pollen, the species richness of pollen, and the species diversity of pollen on pollinators. Each model encompassed the same four underlying structured equations that represent (a) the effects of site type (urban or natural), plant species richness, plant invasive status, pollinator species richness, pollinator rarity, pollinator sex, a pollen abundance on the short‐term specialization measure; (b) the effects of site type, plant species richness, and insect species richness on pollinator rarity; (c) the effects of site type, plant species richness, and insect species richness on plant invasive status; and (d) the effects of pollinator rarity, pollinator sex, plant species richness, and plant invasive status on pollen abundance. We included site as a random effect for equations in models in which the response variable in the first equation was the proportion of conspecific pollen or pollen diversity. We included the date sampled as a random effect in equations in models in which pollen species richness was the response variable of the first equation, because we found that pollen species richness was temporally autocorrelated (see below). We used Gaussian error structures and examined residuals to verify that this was appropriate. Binary categorical variables (site type, plant invasive status, pollinator rarity, and pollinator sex) were encoded numerically as 0/1. All variables were standardized prior to use in models using the normalize function in the BBmisc R package (Bischl et al., 2017). Tests of directed separation were used to validate models and a global goodness of fit was obtained for each model. Estimates reported in the Results section reflect standardized coefficients.

We ran tests for spatial and temporal autocorrelation of the response variables of each structured equation model. To assess spatial autocorrelation, we used the Moran.I function implemented using the ape (Paradis & Schliep, 2019) and MuMin R packages (Bartoń, 2020), and found no autocorrelation. To assess temporal autocorrelation, we implemented continuous‐time first‐order autocorrelation models using the nlme package (Pinheiro et al., 2020). There was temporal autocorrelation for pollen richness, so we included the autocorrelation structure directly in that structured equation model. For the structural equation modeling, we used a dataset that included only pollinators that were reliably identifiable to species and whose sex was decipherable (N = 479; see Table S1).

We evaluated whether pollinators exhibited frequency dependence in foraging by testing for a relationship between preference index PI and plant relative abundance. We used a linear mixed model implemented using the lme4 package in R (Bates et al., 2014), with PI as the response variable, plant relative abundance as the independent variable, and site and plant species as random effects. We tested if pollinator preferences differed between invasive and noninvasive plants and if this depended on site type, using a linear mixed model with PI as the response variable, site type, whether the plant was invasive, and their interaction as independent variables, and site and species as random effects. We determined if species‐level floral specialization differed between site types using a dataset that contained each pollinator species found at each site, d’ of each species as the response variable, and site type as the independent variable. We also determined if site types differed in the number of flowers that were from invasive plants using a generalized linear mixed model with the number of flowers as the response variable, site type and whether the plant from which the flowers came was invasive as fixed effects, site as a random effect, and a Poisson error structure. We examined residuals to verify that the chosen error distributions were appropriate, and we evaluated if fixed factors and their interactions improved models using likelihood ratio tests (LR tests) comparing nested models with and without the factor of interest. Chi‐square and P‐values reported in the Results section reflect the LR tests, and for each model, the estimate (Est.) reported reflects the coefficient of the best model chosen via backward model selection. For generalized linear mixed models, we used summary function in R to evaluate significance of fixed factors and their interactions, and to extract model coefficients. We determined if site types differed in plant or insect species richness or diversity using *t*‐tests.

## 3 RESULTS

### 3.1 Pollen carriage and pollinator specialization

**FIGURE 1:**
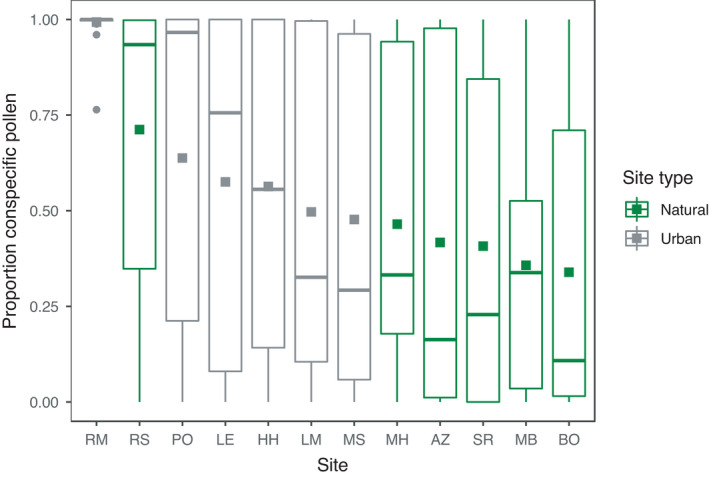
The proportion of conspecific pollen on pollinators across the 12 sites. Natural sites are in green and urban sites are in gray. Data points are actual data points for each pollinator, plots are boxplots, and filled squares within boxplots represent means for each site

**FIGURE 2:**
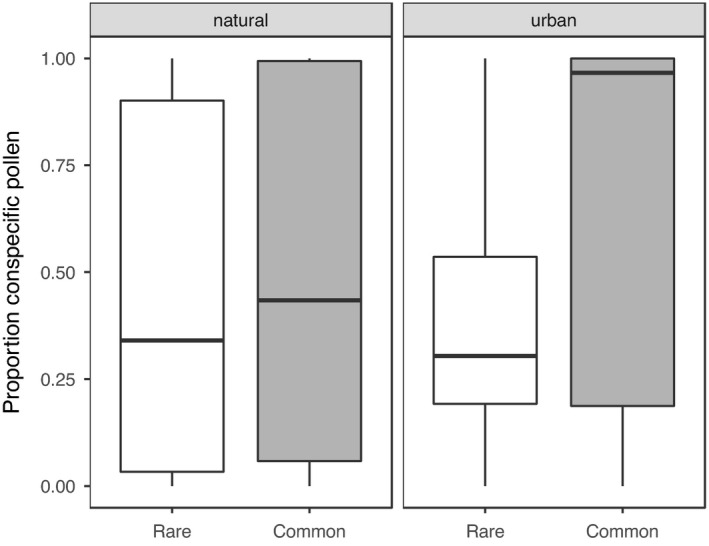
The proportion of conspecific pollen found on rare and common pollinators at natural (left panel) and urban sites (right panel). Points represent actual data points and plots are boxplots

**FIGURE 3:**
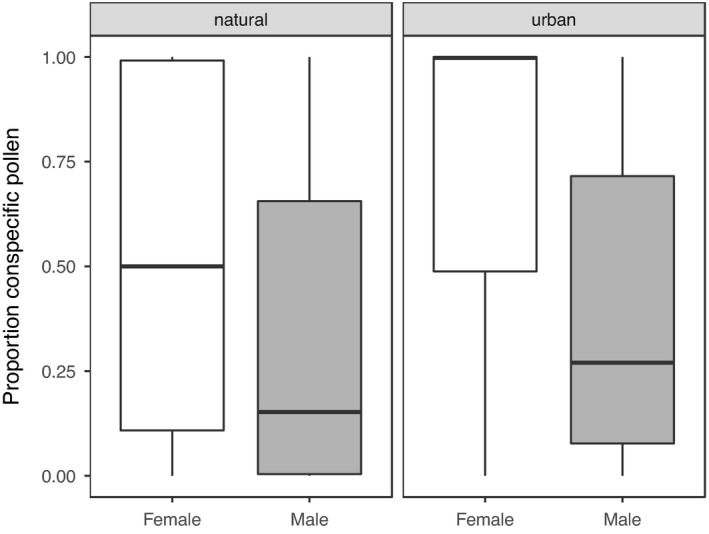
The proportion of conspecific pollen found on female and male pollinators at natural (left panel) and urban sites (right panel). Points represent actual data points and plots are boxplots

**FIGURE 4:**
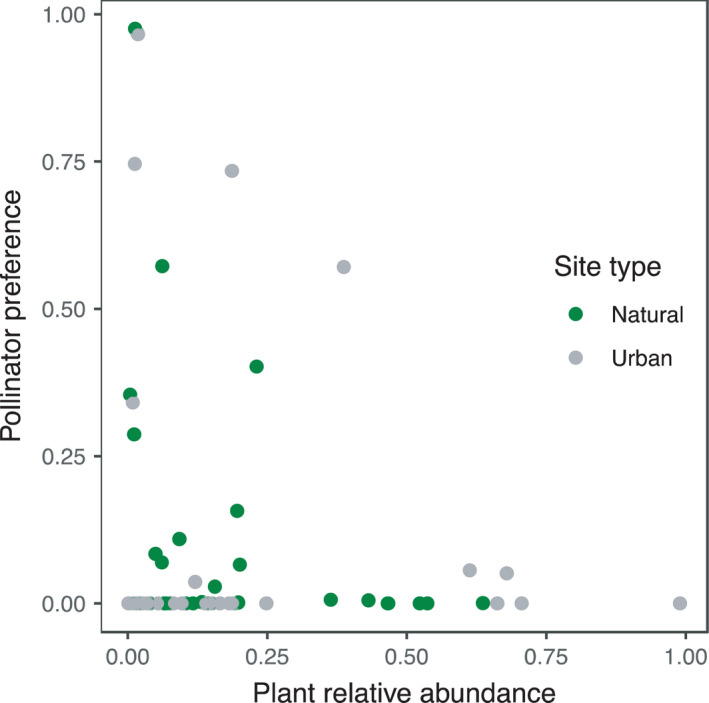
The relationship between plant relative abundance and pollinator preference for each plant species at each site. Plant species at urban sites are in gray and those at natural sites are in green

The 493 insects in our dataset represented 27 different pollinator species, were caught on 43 plant species, and carried pollen from a total of 45 plant species (Table S1). Across urban sites, pollinators carried pollen from a total of 29 species, while across natural sites, they carried pollen from 23 species. Urban pollinators carried on average 1.4 times as much conspecific pollen as pollinators in natural areas (mean urban prop. = 0.64 ± 0.03; mean natural prop. = 0.47 ± 0.03; Figure 1). Common pollinators carried on average 1.4 times as much conspecific pollen as rare pollinators (mean common = 0.6, mean rare = 0.44, Figure 2), and female pollinators carried on average 1.8 times as much conspecific pollen as male pollinators (mean female = 0.64, mean male = 0.36, Figure 3). Pollen species richness was 1.3 times higher in natural sites than in urban sites (mean urban 1.9 ± 0.06; mean natural 2.4 ± 0.08), pollen species diversity was 1.2 times higher in natural sites (mean urban 1.5 ± 0.02; mean natural = 1.8 ± 0.04). There was no difference between site types in species‐level specialization of pollinators (mean urban *d’* = 0.17; mean natural *d’* = 0.13; Est. = 0.03, *t* = 1, *p* = .32). Across sites, pollinators exhibited negative frequency‐dependent foraging such that there was an inverse relationship between plant relative abundance and preference for a given plant species (Est. = −0.34, Chisq = 10.8, *p* = .001; Figure 4).

### 3.2 Plant and pollinator community‐level metrics

Site types did not differ in plant species richness (mean urban = 5, mean natural = 6.3, *t* = 0.87, df = 6.8, *p* = .42) or diversity (mean urban = 3.1, mean natural = 3.9, *t* = 0.67, *p* = .52). Insect species richness was 2.7 times higher in natural sites than in urban sites (mean urban = 2.8; mean natural = 7.5; *t* = 3.5, df = 8.3, *p* = .008), but there was no difference in species diversity (mean urban = 1.5, mean natural = 1.8, *t* = −1.9, df = 6.9, *p* = .10). Floral abundance at the site level was positively associated with the amount of conspecific pollen (Est. = 0.13, Chisq = 8.4, *p* = .004). The number of flowers per plant was higher in urban areas (Est. = 1.9, *z* = 2.0, *p* = .046), and urban sites tended to have a higher total number of flowers, but this trend was not significant (Est. = 1.5, *z* = 1.9, *p* = .06). There was substantial variation among sites in the species richness of invasive plants (Table 1), and urban sites contained more flowers from invasive plants (Est. = 1.2, *z* = 11.2, *p* < .001). The way that floral preference of pollinators differed among invasive and noninvasive taxa depended on site type, such that pollinators preferred noninvasive taxa at natural sites and invasive taxa at urban sites (Interaction Est. = −0.18; Chisq = 4.1, *p* = .045, Figure S2).

### 3.3 Structural equation modeling

**FIGURE 5:**
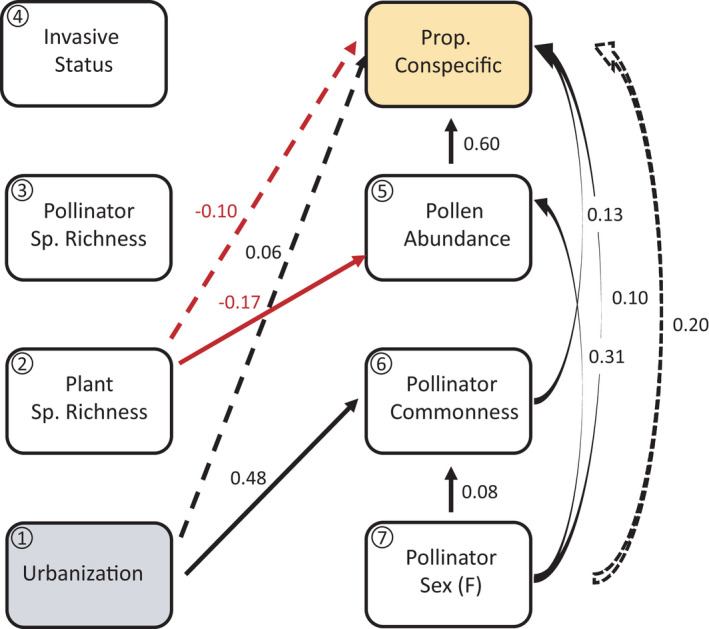
Piecewise structural equation model depicting the relationships among aspects of the plant and pollinator communities and the proportion of pollen carried by pollinators that was from the same plant species as that on which the pollinator was caught (Prop. conspecific). Variables include: (1) whether a site was urban or natural, (2) the plant species richness, (3) pollinator species richness at that site, (4) whether the plant on which the pollinator was caught was invasive, (5) the abundance of pollen on the pollinator, (6) whether the pollinator was one of the two common species, and (7) whether the pollinator was female. Arrows show unidirectional relationships among variables, with black arrows representing positive effects and red arrows representing negative effects. Only significant paths are shown (see Table S2 for model output). Numbers next to arrows represent standardized regression coefficients. Standardized coefficients for indirect effects were calculated by multiplying the coefficients of significant paths, and then summing over indirect paths

Structural equation modeling revealed the direct effects of pollinator rarity and sex, and pollen abundance, on the proportion of conspecific pollen (Figure 5; Table S2). Common pollinators, as well as female pollinators carried higher proportions conspecific pollen, and the amount of grains carried was positively associated with the proportion of conspecific pollen. Pollinator sex also had indirect effects on the proportion of pollen carried that was conspecific, via effects on pollen abundance, as well as because common pollinators were more likely to be female (Figure 5; Table S2). Urbanization had a positive, indirect effect on the proportion of conspecific pollen on pollinators, via differences between common and rare pollinators in the proportion of pollen carried that was conspecific (Figure 5; Table S2). Common pollinators carried more conspecific pollen, and were also more abundant at urban sites (Figure 5; Table S2). Plant species richness had a negative, indirect effect on the proportion of conspecific pollen, which was mediated by pollinators carrying less pollen at sites with higher plant species richness (Figure 5; Table S2).

There were direct effects of pollinator rarity, insect species richness, and pollen abundance on the species richness of pollen on pollinators (Figure S3; Table S2). Common pollinators carried pollen from fewer plant species, and pollinators carried pollen from fewer plant species at sites with higher insect species richness. Pollinators that carried greater amounts of pollen carried pollen from fewer plant species. Plant species richness had a positive indirect effect on pollen species richness, via effects on the total amount of pollen carried (Figure S3; Table S2). Pollinators carried less pollen at sites with higher plant species richness, and the species richness of pollen on pollinators was lower when they carried more total pollen. Plant invasiveness had a negative indirect effect on pollen species richness, via effects on the total amount of pollen on pollinators (Figure S3; Table S2). Pollinators carried more pollen from invasive plants, and pollinators that carried more pollen carried pollen from fewer plant species. Pollinator sex had a negative indirect effect on pollen species richness via effects on pollen abundance and pollen commonness. Female pollinators carried more pollen, and pollinators that carried more pollen carried pollen from fewer plants species (Figure S3; Table S2). In addition, female pollinators were more likely to be common species, and common pollinators carried pollen from fewer plant species (Figure S3; Table S2).

There were direct effects of pollinator rarity and pollen abundance on the species diversity of pollen carried by pollinators. Pollen species diversity was lower on common pollinators and was higher on pollinators that carried more pollen (Figure S4; Table S2). Plant species richness had a negative indirect effect on pollen species diversity, via effects on pollen abundance (Figure S4; Table S2). Plant invasiveness had a positive indirect effect on pollen species diversity, also via effects on pollen abundance (Figure S4; Table S2). Pollinator sex had a positive indirect effect on pollen species diversity via effects on pollen abundance (Figure S4; Table S2).

## 4 DISCUSSION

Our finding positive effects of urbanization on the proportion of conspecific pollen carried by pollinators suggests that pollinators exhibited greater short‐term specialization in urban areas. This finding was corroborated by the higher species richness of pollen on pollinators from natural sites. Our findings also suggest strong potential differences between common and rare pollinators, and female and male pollinators, in effects on plant fitness. In addition, our results point toward several ecological drivers of variation in short‐term specialization, including interspecific competition among pollinators, the abundance and diversity of food resources, and frequency‐dependent foraging.

Effects of urbanization on short‐term specialization were indirect, and were mediated by common pollinators carrying higher proportions of conspecific pollen. Relative to natural sites, average conspecific pollen amounts increased by 54% and 23% for the common pollinators *A*. *mellifera* and *B*. *vosnesenskii* in urban sites. These results suggest that given the pollinator assemblages present at urban sites, pollinator foraging choices may largely benefit plant fitness. It is worth noting that, for self‐incompatible plants, whether or not these high proportions of conspecific pollen facilitate plant seed production depends on whether the pollen carried is deposited on different individuals of the same plant species. Honeybees have been observed to visit many flowers on the same plant individual, resulting primarily in the transfer of self‐pollen (Ivey et al., 2003). This suggests that the higher conspecific pollen loads may not always translate into higher plant fitness, and is worth investigating.

In addition to common pollinators, female pollinators carried higher proportions of conspecific pollen. While this did not drive differences between urban and natural sites, it has implications for plant reproductive success over the growing season. Especially for bees, males tend to be abundant later in the growing season and may be especially important pollinators of late‐blooming plants. Insect pollinators are often sexually dimorphic and differ in traits that can influence pollen transfer, such as body size (del Castillo & Fairbairn, 2011) and tongue length (Wolf & Moritz, 2014). Males tend to forage less than females (Michener, 2000) but have longer flower handling times and be more likely to leave patches after a few flower visits (Ostevik et al., 2010), which could reduce self‐pollen transfer. Similarly, male bees may have higher pollen transfer efficiency, in terms of the amount of pollen removed from anthers and deposited on stigmas (Tang et al., 2019, but see Ogilvie & Thomson, 2015). In contrast to these studies, we find no evidence that pollination by males would lead to higher plant reproductive success. Males carried lower proportions of conspecific pollen than females. Therefore, future work should examine the relative reproductive benefits to plants of being visited by male bees, in terms of the proportion of conspecific pollen carried and pollen transfer distances. This would aid our understanding of the importance of male bees to pollination, especially in urban areas where floral patches may be smaller and among‐patch pollen transfer may be important to prevent inbreeding.

Our data support the widely held hypothesis based on ecological theory that greater interspecific competition leads to greater species‐level specialization in resource use (Lawlor & Smith, 1976). Across sites, pollinator species richness had a negative effect on the species richness of pollen carried by pollinators, suggesting that interspecific competition may have driven niche partitioning among species. These findings are consistent with other studies that found that resource partitioning among pollinators increases with competition. For example, Fründ et al. (2013) found that in the presence of a competitor, bee species switched to less‐desirable resources. Similarly, Spiesman and Gratton (2016) showed that modularity of pollination networks increases with higher pollinator species richness, suggesting competition‐mediated effects on foraging choices of pollinators. These studies support the prediction that urbanization should relax interspecific competition and lead to lower specialization on individual plant resources due to lower pollinator species diversity. However, in our case, while there were positive effects of pollinator species richness on specialization across all sites, there was overall higher specialization of pollinators in urban areas despite having lower insect species richness. This suggests that other factors that varied among site types such as intraspecific competition or the abundance of food resources may have influenced pollinator foraging choices.

Floral abundance at the site level was positively associated with conspecific pollen amount, there were more flowers per plant in urban areas, and there was a trend toward higher total floral abundance in urban areas. Since there was no difference in plant species richness or diversity between site types, it follows that pollinators would carry more conspecific pollen in urban areas. Why might floral abundance predict short‐term specialization? Most pollinators do not have fixed affinities for certain plants (Waser et al., 1996) and will continue to forage on flowers of particular species when those flowers are sufficiently abundant and rewarding that travel costs incurred by passing up flowers of other species are low (Heinrich, 1979; Waser, 1986). In addition, pollinators spend longer on flowers if there is more, or higher quality nectar present (Thomson, 1986), which might facilitate the accumulation of more pollen. In contrast, pollinators are more likely to switch plant species when they consistently encounter flowers of a particular species that are rewardless (Grüter et al., 2011). It is possible that pollinators experience rewarding flowers more often than pollinators at natural sites because of the higher number of flowers in urban sites. It is also possible that floral reward is greater in urban sites due to an overall lower abundance of pollinators, or more favorable ecological conditions such as moisture levels that promote the production of more flowers or flowers with greater rewards.

Another potential driver that may contribute to the higher specialization we observed for urban pollinators is the amount of energy pollinators may need to spend acquiring sufficient resources in different environments. The importance of pollinator movement to the acquisition of sufficient resources is largely unknown (Harrison & Winfree, 2015), but pollinators have been observed to spend longer amounts of time in urban flower patches than in large continuous countryside populations (Andrieu et al., 2009). Urban landscapes are characterized by large regions of inhospitable habitat over which pollinators may need to travel to reach food resources. This longer time spent in urban fragments presumably allows pollinators to recoup energetic costs of travel among patches. After arriving in an urban patch, pollinators may be likely to continue foraging on the same species of plant and not expend energy learning to manipulate alternative floral types. The extent to which pollinators move among urban fragments in our study area would be a valuable future research direction.

Our findings have implications for native plant species coexistence in urban habitat fragments and natural areas. The negative frequency‐dependent foraging exhibited by pollinators suggests that pollinator foraging favors the reproduction of rare plant species, although there were some rare species that received few visits. We also found evidence that pollinators prefer noninvasive taxa in natural sites, which could dampen the negative effects of invasive plants on native plants in those sites. However, pollinators preferred to forage on invasive plants at urban sites. Many studies have reported positive relationships between urbanization and invasive plant abundance (Bradley & Mustard, 2006; George et al., 2009; Seabloom et al., 2006), as well as preferences for invasive taxa (Stout & Tiedeken, 2017). The stronger preference invasive plants in the urban areas examined in this study suggests that native plant species may receive fewer visits by pollinators than invasive species. Therefore, the persistence of native plants in urban areas may depend on continued augmentation of native flora and removal of invasive species.

Overall, the conspecific pollen proportions found on pollinators suggest that there is a greater potential for conspecific pollen transfer among plants in urban areas than in natural areas. Furthermore, plant reproductive success in urban areas may depend disproportionately on common species, which carried higher proportions of conspecific pollen. This would be consistent with recent work showing that provisioning of ecosystem services depends less on species richness than on services provided by a few common species (Winfree et al., 2015). An important caveat of these implications is that we did not explicitly measure flower constancy, so we cannot know if pollinators moved among flowers of the same individual plant or among flowers of different individuals. Self‐incompatible plants cannot produce seeds unless they receive pollen from a different conspecific individual (Castric & Vekemans, 2004). Therefore, it will be worthwhile to explore whether the greater conspecific pollen proportions found on urban pollinators are reflective of visits to single plant individuals or several different plants of the same species.

## AUTHOR CONTRIBUTIONS

**Sevan Suni:** Conceptualization (lead); Data curation (lead); Formal analysis (lead); Funding acquisition (lead); Investigation (equal); Methodology (lead); Project administration (equal); Resources (lead); Software (lead); Supervision (supporting); Validation (lead); Visualization (lead); Writing – original draft (lead); Writing – review & editing (lead). **Erin Hall:** Data curation (equal); Investigation (supporting); Methodology (supporting); Project administration (supporting); Writing – review & editing (supporting). **Evangelina Bahu:** Data curation (equal); Investigation (supporting); Methodology (supporting); Project administration (supporting); Writing – review & editing (supporting). **Hannah Hayes:** Data curation (supporting); Formal analysis (supporting); Validation (supporting); Writing – review & editing (supporting).

### OPEN RESEARCH BADGES

This article has earned an Open Data Badge for making publicly available the digitally‐shareable data necessary to reproduce the reported results. The data is available at 10.5281/zenodo.5213100.

## Supporting information

Supplementary Material

## Data Availability

Data and custom scripts are available on Zenodo.com (https://doi.org/10.5281/zenodo.5213100).
