# Supplementary material for: Urbanization increases floral specialization of pollinators
Source: Ecol Evol. 2022 Mar 7;12(3):e8619. doi: 10.1002/ece3.8619 (PMC8901868; doi:10.1002/ece3.8619)
Supplement: Supplementary file 1 — Supplementary Material [file ECE3-12-e8619-s001.docx]

**Supplementary Information for “Urbanization Increases Floral Fidelity of Pollinators”,**

**by Sevan Suni, Erin Hall, Evangelina Bahu, and Hannah Hayes**

**Tables and Figures Included:**

**Table S1 –** Pages 2 - 5

**Table S2 –** Page 6 - 7

**Figure S1 –** Page 8

**Figure S2 –** Page 9

**Figure S3 –** Page 10

**Figure S4 –** Page 11

**Table S1.** Pollinator species, whether the site was urban (U) or natural (N), the site abbreviation, and the number of grains of pollen from each plant species found on that pollinator. Plants included reflect those that were found across all sites.

**Table S2**

| **Model** | **Response** | **Predictor** | **Estimate** | **SE** | **DF** | **Crit. Value** | **P-value** | **Std. Estimate** |
| --- | --- | --- | --- | --- | --- | --- | --- | --- |
| 1 | Prop. conspecific | Pollen Abundance | 0.75 | 0.04 | 450 | 312 | 0.000 | 0.60 |
|  |  | Pollinator Sex | 0.09 | 0.03 | 455 | 8.3 | 0.004 | 0.10 |
|  |  | Pollinator Commonness | 0.13 | 0.04 | 452 | 10 | 0.002 | 0.13 |
|  |  | Site Type | 0.17 | 0.13 | 8.4 | 1.9 | 0.205 | 0.21 |
|  |  | Invasive Status | -0.07 | 0.04 | 348 | 3.2 | 0.077 | -0.08 |
|  |  | Plant Sp. Richness | -0.16 | 0.16 | 8.3 | 1.0 | 0.335 | -0.10 |
|  |  | Insect Sp. Richness | 0.20 | 0.19 | 7.9 | 1.1 | 0.335 | 0.16 |
|  | Pollen Abundance | Pollinator Commonness | 0.00 | 0.04 | 68 | 0.0 | 0.994 | 0.00 |
|  |  | Pollinator Sex | 0.23 | 0.03 | 343 | 46 | 0.000 | 0.31 |
|  |  | Plant Sp. Richness | -0.21 | 0.06 | 9.8 | 10.8 | 0.008 | -0.17 |
|  |  | Invasive Status | 0.07 | 0.03 | 35.1 | 4.0 | 0.053 | 0.10 |
|  | Pollinator Commonness | Site Type | 0.40 | 0.17 | 8.2 | 5.4 | 0.047 | 0.48 |
|  |  | Plant Sp. Richness | 0.11 | 0.22 | 8.3 | 0.3 | 0.626 | 0.07 |
|  |  | Pollinator Sex | 0.08 | 0.04 | 458 | 4.7 | 0.031 | 0.08 |
|  |  | Insect Sp. Richness | -0.05 | 0.27 | 8.1 | 0.0 | 0.843 | -0.04 |
|  | Pollinator Sex | Site Type | -0.08 | 0.18 | 8.3 | 0.2 | 0.673 | -0.09 |
|  |  | Plant Sp. Richness | 0.09 | 0.22 | 8.5 | 0.1 | 0.709 | 0.05 |
|  |  | Insect Sp. Richness | -0.18 | 0.27 | 8.1 | 0.5 | 0.520 | -0.14 |
|  | Invasive Status | Site Type | -0.28 | 0.33 | 8.1 | 0.7 | 0.419 | -0.29 |
|  |  | Plant Sp. Richness | -0.34 | 0.41 | 8.1 | 0.7 | 0.440 | -0.18 |
|  |  | Insect Sp. Richness | 0.15 | 0.51 | 8.0 | 0.1 | 0.771 | 0.11 |
| 2 | Pollen richness | Site Type | -0.07 | 0.04 | 8 | -2.0 | 0.085 | -0.21 |
|  |  | Sampling Date | -0.13 | 0.04 | 8 | -3.2 | 0.013 | -0.26 |
|  |  | Pollinator Sex | 0.01 | 0.02 | 447 | 0.8 | 0.400 | 0.04 |
|  |  | Commonness | -0.09 | 0.02 | 447 | -3.5 | 0.001 | -0.22 |
|  |  | Invasive Status | 0.00 | 0.02 | 447 | 0.0 | 0.990 | 0.00 |
|  |  | Plant Sp. Richness | 0.05 | 0.04 | 447 | 1.0 | 0.312 | 0.07 |
|  |  | Insect Sp. Richness | -0.16 | 0.05 | 447 | -3.0 | 0.003 | -0.33 |
|  |  | Total | -0.06 | 0.02 | 447 | -2.5 | 0.011 | -0.11 |
|  | Total | Commonness | -0.02 | 0.05 | 449 | -0.4 | 0.673 | -0.02 |
|  |  | Pollinator Sex | 0.20 | 0.03 | 449 | 6.0 | 0.000 | 0.27 |
|  |  | Plant Sp. Richness | -0.16 | 0.08 | 449 | -2.0 | 0.045 | -0.13 |
|  |  | Invasive Status | 0.08 | 0.04 | 449 | 2.1 | 0.034 | 0.11 |
|  |  | Sampling Date | 0.14 | 0.07 | 9 | 1.9 | 0.086 | 0.13 |
|  | Commonness | Site Type | 0.45 | 0.22 | 8 | 2.1 | 0.072 | 0.54 |
|  |  | Plant Sp. Richness | 0.01 | 0.23 | 450 | 0.0 | 0.966 | 0.01 |
|  |  | Insect Sp. Richness | 0.10 | 0.33 | 450 | 0.3 | 0.766 | 0.08 |
|  |  | Pollinator Sex | 0.05 | 0.02 | 450 | 2.2 | 0.026 | 0.05 |
|  |  | Sampling Date | 0.13 | 0.27 | 8 | 0.5 | 0.645 | 0.10 |
|  | Pollinator Sex | Site Type | -0.12 | 0.15 | 8 | -0.8 | 0.454 | -0.13 |
|  |  | Plant Sp. Richness | 0.02 | 0.18 | 451 | 0.1 | 0.934 | 0.01 |
|  |  | Insect Sp. Richness | 0.02 | 0.24 | 451 | 0.1 | 0.930 | 0.02 |
|  |  | Sampling Date | 0.43 | 0.19 | 8 | 2.2 | 0.058 | 0.30 |
|  | Invasive Status | Site Type | -0.65 | 0.72 | 8 | -0.9 | 0.394 | -0.67 |
|  |  | Plant Sp. Richness | 1.36 | 0.53 | 451 | 2.6 | 0.010 | 0.75 |
|  |  | Insect Sp. Richness | -1.61 | 0.97 | 451 | -1.7 | 0.098 | -1.11 |
|  |  | Sampling Date | -0.98 | 0.89 | 8 | -1.1 | 0.302 | -0.64 |

**Table S2 – continued on next page**

**Table S2 – continued from previous page**

| **Model** | **Response** | **Predictor** | **Estimate** | **SE** | **DF** | **Crit. Value** | **P-value** | **Std. Estimate** |
| --- | --- | --- | --- | --- | --- | --- | --- | --- |
| 3 | Pollen Species Diversity | Total | 0.07 | 0.03 | 291 | 7.3 | 0.007 | 0.14 |
|  |  | Pollinator Sex | -0.04 | 0.02 | 296 | 2.7 | 0.103 | -0.09 |
|  |  | Commonness | -0.12 | 0.03 | 293 | 19.6 | 0.000 | -0.30 |
|  |  | Site Type | -0.11 | 0.07 | 8.4 | 2.4 | 0.159 | -0.31 |
|  |  | Invasive Status | 0.02 | 0.03 | 200 | 0.3 | 0.588 | 0.04 |
|  |  | Plant Sp. Richness | 0.08 | 0.09 | 9.0 | 0.8 | 0.401 | 0.11 |
|  |  | Insect Sp. Richness | -0.16 | 0.11 | 7.7 | 1.9 | 0.202 | -0.28 |
|  | Total | Commonness | -0.04 | 0.05 | 38.6 | 0.5 | 0.476 | -0.04 |
|  |  | Pollinator Sex | 0.19 | 0.04 | 231 | 18.3 | 0.000 | 0.25 |
|  |  | Plant Sp. Richness | -0.20 | 0.09 | 12.9 | 4.9 | 0.045 | -0.13 |
|  |  | Invasive Status | 0.10 | 0.04 | 26.0 | 4.8 | 0.038 | 0.14 |
|  | Commonness | Site Type | 0.41 | 0.19 | 8.1 | 4.7 | 0.061 | 0.46 |
|  |  | Plant Sp. Richness | 0.11 | 0.24 | 8.6 | 0.2 | 0.656 | 0.06 |
|  |  | Insect Sp. Richness | -0.12 | 0.29 | 8.0 | 0.2 | 0.681 | -0.09 |
|  |  | Pollinator Sex | 0.04 | 0.04 | 298 | 1.0 | 0.317 | 0.05 |
|  | Pollinator Sex | Site Type | -0.12 | 0.20 | 8.2 | 0.4 | 0.567 | -0.13 |
|  |  | Plant Sp. Richness | 0.22 | 0.26 | 9.0 | 0.7 | 0.424 | 0.11 |
|  |  | Insect Sp. Richness | -0.17 | 0.31 | 8.1 | 0.3 | 0.610 | -0.12 |
|  | Invasive Status | Site Type | -0.36 | 0.34 | 8.0 | 1.1 | 0.317 | -0.37 |
|  |  | Plant Sp. Richness | -0.42 | 0.43 | 8.2 | 1.0 | 0.356 | -0.21 |
|  |  | Insect Sp. Richness | -0.06 | 0.53 | 8.0 | 0.0 | 0.914 | -0.04 |

**Table S2.** Output from three piecewise structural equation models depicting relationships among aspects of the plant and pollinator communities and the proportion of pollen on pollinators that was conspecific, the species richness of pollen carried by pollinators, and the species diversity of pollen on pollinators. See Figures 2, S2, and S3 for path diagrams of model output.

**Figure S1**

**Figure S1.** Sampling locations throughout the San Francisco Bay Area in the state of California, USA. Circles represent natural sites and squares represent urban sites. Note that two urban sites were close together and are visible as a slightly darker square. The star on the grey map (bottom left) represents the sampling area within the United States.

**Figure S2**. Pollinator preference index (PI) for invasive (grey) and non-invasive (white) plants at urban and natural sites. Points represent actual data points and outlines represent boxplots.

**Figure S3.** Piecewise structural equation model depicting the relationships among aspects of the plant and pollinator communities and the species richness of pollen carried by pollinators. Variables include: (1) whether a site was urban or natural, (2) the plant species richness, and (3) pollinator species richness at that site, (4) whether the plant on which the pollinator was caught was invasive (5) the abundance of pollen on the pollinator, (6) whether the pollinator was one of the two common species, and (7) whether the pollinator was a female. Arrows show unidirectional relationships among variables, with black arrows representing positive effects and red arrows representing negative effects. Only significant paths are shown (see Table S2 for model output). Numbers next to arrows represent standardized regression coefficients. Standardized coefficients for indirect effects were calculated by multiplying the coefficients of significant paths, and then summing over indirect paths.

**Figure S4.** Piecewise structural equation model depicting the relationships among aspects of the plant and pollinator communities and the species diversity of pollen on pollinators. Variables include: (1) whether a site was urban or natural, (2) the plant species richness, and (3) pollinator species richness at that site, (4) whether the plant on which the pollinator was caught was invasive (5) the abundance of pollen on the pollinator, (6) whether the pollinator was one of the two common species, and (7) whether the pollinator was a female. Arrows show unidirectional relationships among variables, with black arrows representing positive effects and red arrows representing negative effects. Only significant paths are shown (see Table S2 for model output). Numbers next to arrows represent standardized regression coefficients. Standardized coefficients for indirect effects were calculated by multiplying the coefficients of significant paths, and then summing over indirect paths.
